# Association of Long-Term Care Risk with Nonresponse to the Annual Frailty Screening Program in Older Adults in Japan: A Retrospective Cohort Study

**DOI:** 10.31662/jmaj.2025-0092

**Published:** 2025-09-12

**Authors:** Kazumasa Nishida, Reina Taguchi, Rumiko Tsuchiya-Ito, Tomoki Ishikawa, Satomi Kitamura, Masao Iwagami, Shinji Hattori, Shota Hamada

**Affiliations:** 1 Research Department, Institute for Health Economics and Policy, Association for Health Economics Research and Social Insurance and Welfare, Tokyo, Japan; 2 Department of Health, Labor and Welfare Policy Research, JMA Research Institute Inc, Tokyo, Japan; 3 Research Department, Dia Foundation for Research on Ageing Societies, Tokyo, Japan; 4 Department of Health Services Research, Institute of Medicine, University of Tsukuba, Ibaraki, Japan; 5 Department of Home Care Medicine, Graduate School of Medicine, The University of Tokyo, Tokyo, Japan

**Keywords:** nonresponse, long-term care risk, frailty, Kihon Checklist, annual frailty screening program

## Abstract

**Introduction:**

This study aimed to determine the association between long-term care (LTC) risk and nonresponse to a subsequent survey.

**Methods:**

Community-dwelling older adults aged ≥75 years without certified care needs residing in Hachioji City, Tokyo, who participated in the annual frailty screening program, were evaluated. Among the fiscal year (FY) 2020 survey respondents, those invited to the FY 2021 survey were included. The exposures of interest were LTC risk assessed using the seven domains of the Kihon Checklist (KCL) and frailty status in the FY 2020 survey. Frailty status was categorized based on total KCL scores. The outcome of interest was nonresponse to the FY 2021 survey. We conducted multivariable logistic regression analysis to evaluate these associations.

**Results:**

Among 35,425 participants, 9,456 (26.7%) did not respond to the subsequent survey. Among seven KCL domains, activities of daily living limitation (adjusted odds ratio, 95% confidence interval 1.38, 1.27-1.50), low physical strength (1.17, 1.10-1.26), isolation (1.32, 1.22-1.44), memory decline (1.23, 1.17-1.30), and depressive mood (1.10, 1.04-1.16) were associated with nonresponse to the subsequent survey. In addition, frailty status was associated with nonresponse in a dose-responsive manner (prefrailty: 1.19, 1.12-1.26; frailty: 1.63, 1.53-1.73).

**Conclusions:**

Although the annual frailty screening program aimed to identify those with high LTC risk, older adults with LTC risk were less likely to respond to the survey. Thus, conventional survey methods may need to be modified or different approaches may need to be adopted to identify older adults with high LTC risk.

## Introduction

The identification of older adults at risk of long-term care (LTC) is essential as early interventions can prevent disability and LTC utilization ^(1), (2)^ Although older adults with LTC risk factors including frailty are at higher risk of disability and mortality ^(3), (4), (5), (6), (7)^, they can recover their function with appropriate interventions ^(8), (9), (10)^. In Japan, an annual nationwide frailty screening program using a questionnaire sent by mail is implemented to identify community-dwelling older adults at high risk for LTC ^(11)^. This program has been governed by municipalities as insurers under the Japanese LTC insurance system since 2006 and continues in some municipalities at their discretion ^(12)^. Japanese municipalities need to establish the number of older adult residents at risk of requiring care and construct a strategy to prevent the acceleration of their frailty and disabilities. However, this type of survey always carries the risk of excluding older adults who do not or cannot respond to it. Reducing nonresponse in questionnaire surveys is crucial because a high nonresponse rate decreases the validity of the survey ^(13), (14)^. Accordingly, some studies have focused on dropouts in cohort studies ^(15), (16), (17)^ and explored strategies for improving response or participation rates, including monetary incentives or recorded delivery ^(18), (19)^. However, nonresponse to repeated cross-sectional questionnaire surveys in older adults, such as the annual frailty screening program mentioned above, has not been well reported.

Some cohort studies have found that nonresponses and dropouts were more prevalent among those with cognitive or physical decline or social vulnerabilities ^(14), (20), (21)^. If this is the case in the annual frailty screening program, the program might overlook high-risk populations for LTC, even though they are intended targets for identification. Therefore, these findings should be verified through annual frailty screening programs for community-dwelling older adults. The annual frailty screening program uses the 25-item Kihon Checklist (KCL) to assess physical and cognitive decline and social vulnerabilities leading to the need for LTC ^(22)^. In addition, overall frailty status is assessed using the total KCL score across the seven domains ^(23)^. Practically, it is difficult to directly investigate the characteristics of individuals who do not respond to a survey; instead, we aimed to evaluate the longitudinal association of LTC risks assessed using the KCL with nonresponses in the subsequent year using survey data from two consecutive years.

## Materials and Methods

### Data source and study participants

We used data from the annual frailty screening program conducted by Hachioji City, Tokyo, with fiscal year (FY) 2020 as the baseline survey and FY 2021 as the subsequent survey. This program was conducted for community-dwelling older adults aged ≥75 years who were not certified to have LTC needs. The prevalence of frailty in patients aged 65-74 years (4.0%) was much lower than that in patients older than 75 years (aged 75-84 years, 16.2%; older than 85 years, 34.0%) ^(24)^. In Japan, the Medical Insurance Systems shift to the Late-Stage Elderly Medical Care System at the age of 75 years, at which point the number of people with LTC needs rises ^(25)^. Therefore, people older than 75 years were considered the target population for screening for LTC needs. The survey items included sex, age, chronic diseases, living area, and KCL items ^(22)^. The annual frailty screening program in FY 2020 included 35 items, and this increased to 50 in the FY 2021 program from the addition of more detailed variables, such as psychological and social aspects. The response rates were 81.9% in FY 2020 and 64.5% for FY 2021. In addition, we used LTC certification data and LTC insurance premium data, which were linked with the annual frailty screening program data. The LTC certification data were used to identify individuals who had already certified LTC needs between the FY 2020 and FY 2021 surveys. The LTC insurance premium data was used to determine the household income.

Participants in this study were those who were mailed to both the FY 2020 and FY 2021 surveys of the annual frailty screening program. If the participants in the baseline survey were newly certified for LTC needs in a subsequent survey, they were excluded from the study.

### Exposure

The exposures of interest were the LTC risk and frailty status assessed using the KCL in the baseline survey. Briefly, the KCL includes seven domains: activities of daily living (ADL), physical strength, nutrition, oral function, isolation, memory, and mood. Each domain has its own cut-off point for LTC risks. ADL limitation was determined if at least three of the five items were applicable (questions 1-5) ^(26)^. Low physical strength was defined as the applicability of at least three items (questions 6-10). Malnutrition was defined as the presence of both items (questions 11-12). Oral dysfunction was defined as the presence of at least two of the three items (questions 13-15). Isolation was defined as the presence of one item (question 16). Memory decline was defined as the presence of at least one of three items (questions 18-20). Finally, depressive mood was defined as the presence of at least two of five items (questions 21-25) ^(27)^. Frailty status was also evaluated using the KCL as previously described ^(23)^. Frailty status was classified into three levels: frailty, scores of ≥8 points; pre-frailty, scores of 4-7 points; and robust, scores of ≤3 points ^(23)^. The sensitivity and specificity of the KCL to assess people who were “pre-frailty” were 70.3% and 78.3%, respectively, and those who were “frailty” were 89.5% and 80.7%, respectively ^(23)^.

### Outcome

The outcome of interest was nonresponse to the FY 2021 survey. Response to the FY 2021 survey was determined based on the participant’s survey response history. Thus, respondents were individuals who completed the survey in both FY 2020 and FY 2021, whereas nonrespondents were individuals who completed only the survey in FY 2020 and not in FY 2021.

### Covariates

We chose five variables as covariates: sex (female/male), age, household income (standards or lower/higher than standards), chronic diseases (heart disease, diabetes, hypertension, joint pain, fracture, renal failure, respiratory disease, and cerebrovascular disease), and living areas (21 areas). Household income was classified according to the LTC insurance premium levels, which comprised 14 categories in the study setting. Each municipality sets standard fees, and the premiums depend on each individual’s ability to pay, according to the residential taxation status of individuals and households. In this study, household income is categorized into two levels: “higher than standards” and “standards or lower.” In the study setting, level 5, which means “the individual was exempt from residential taxation, but some member of the household paid it,” was classified into the “standards or lower” category.

### Statistical analysis

The baseline characteristics in the FY 2020 survey were compared between the respondents and nonrespondents in the FY 2021 survey. Continuous values were compared using the t-test, and categorical values were compared using the chi-square test. Logistic regression analysis was used to evaluate the associations of the seven KCL domains or frailty status separately with response to the FY 2021 survey by estimating odds ratios (ORs) with their 95% confidence intervals (CIs). In the multivariable logistic regression, age was treated as a continuous variable, whereas the other variables were treated as categorical variables. All hypothesis tests employed a two-sided statistical significance level of 0.05. All statistical analyses were performed using Stata version 17 (Stata Corp, College Station, TX, USA).

## Results

**Figure 1.**
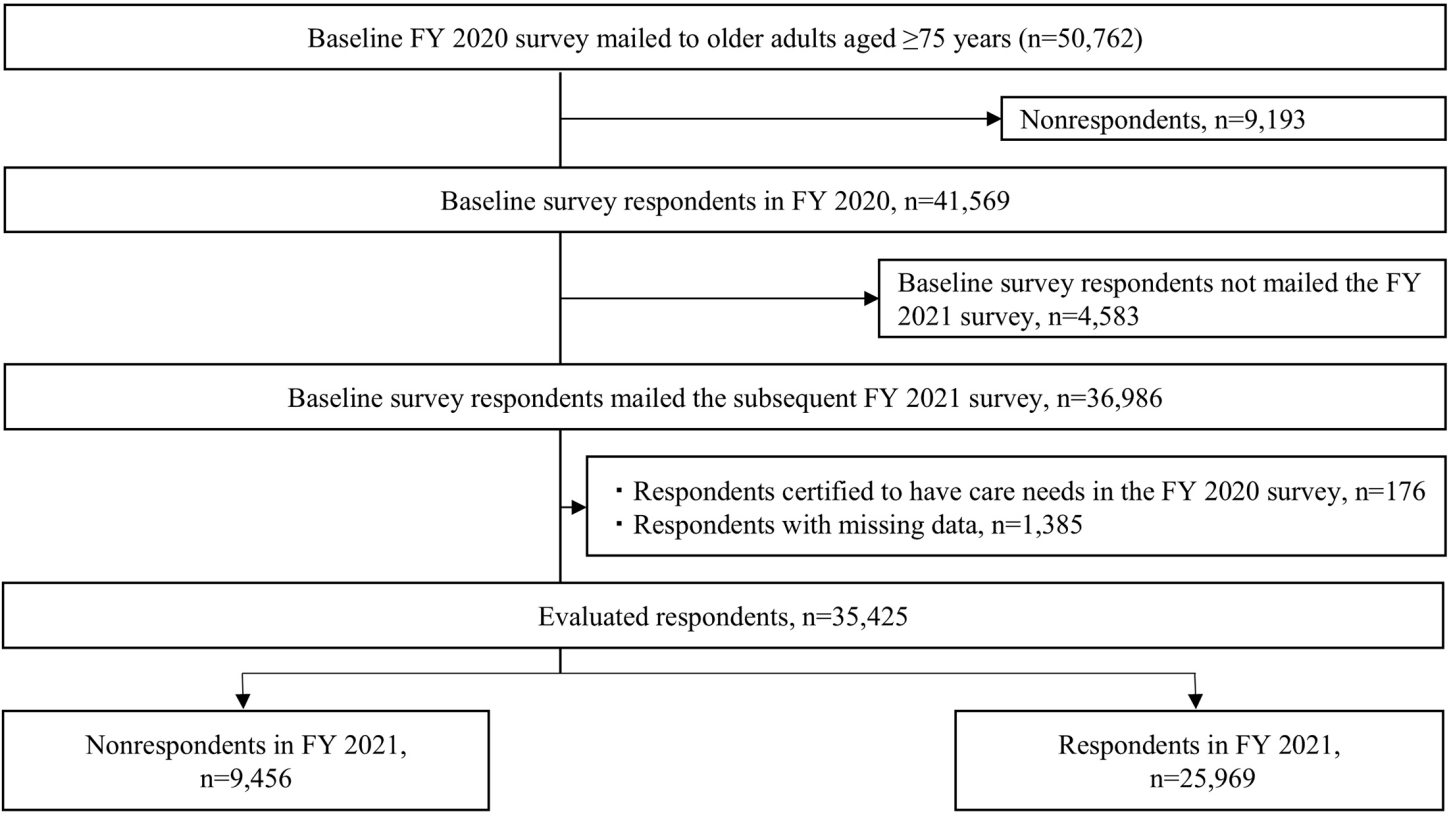
Flow chart of participant selection. FY: fiscal year.

Of the 50,762 older adults aged ≥75 years who completed the FY 2020 survey, 41,569 responded to the FY 2021 survey. Among them, 4,583 respondents in FY 2020 were not mailed the subsequent survey in FY 2021 because they were identified to have moved outside the city, were already certified as LTC beneficiaries, or had died before FY 2021. Thus, 36,986 older adults were mailed the subsequent survey in FY 2021. After excluding 1,561 individuals who were certified as requiring LTC in FY 2020 (n = 176) and were missing data for the KCL items or all covariates (n = 1,385), 35,425 individuals remained and were included in the analysis. Among them, 25,969 (73.3%) individuals responded to the FY 2021 survey. The participant selection flow chart is shown in Figure 1.

The characteristics of study participants are presented in Table 1. Compared with the respondents, the nonrespondents involved a higher proportion of female participants (nonrespondents: 53.1% vs. respondents: 51.6%), participants with household income categorized into standards or lower (58.3% vs. 53.1%), and older participants (average age: 80.2 ± 4.1 vs. 79.9 ± 3.9 years old). Regarding chronic diseases, fractures (2.2% vs. 1.6%) and respiratory diseases (5.9% vs. 5.1%) were more common in nonrespondents, but there were no significant differences in other diseases. The distribution of participants for each LTC risk domain and by frailty status is also shown in Table 1. The percentages of ADL limitation (nonrespondents: 12.5% vs. respondents: 7.6%), low physical strength (18.8% vs. 14.0%), malnutrition (1.8% vs. 1.4%), oral dysfunction (22.7% vs. 19.8%), isolation (12.2% vs. 7.7%), memory decline (37.9% vs. 30.7%), depressive mood (37.1% vs. 31.9%), and frailty (32.5% vs. 24.0%) were higher in nonrespondents than in respondents. In addition, the prevalence of LTC risks for those certified as requiring LTC (n = 176, excluded from selecting study participants) was higher than that in the nonresponse group (Appendix 1).

**Table 1.** Characteristics of Study Participants.

Variable	Total (n = 35,425)	Nonrespondents (n = 9,456)	Respondents (n = 25,969)	p Value
	n (%)	n (%)	n (%)	
Sex				0.009
Male	17,004 (48.0)	4,431 (46.9)	12,573 (48.4)	
Female	18,421 (52.0)	5,025 (53.1)	13,396 (51.6)	
Age (years)				<0.001
Average (SD)^a^	80.0 (3.9)	80.2 (4.1)	79.9 (3.9)	<0.001
Median (IQR)	79 (77-82)	79 (77-83)	79 (77-82)	<0.001
Household income				<0.001
Higher than standards	16,138 (45.6)	3,947 (41.7)	12,191 (46.9)	
Standards or lower	19,287 (54.4)	5,509 (58.3)	13,778 (53.1)	
Chronic diseases				
Heart disease	4,246 (12.0)	1,098 (11.6)	3,148 (12.1)	0.191
Diabetes	4,690 (13.2)	1,305 (13.8)	3,385 (13.0)	0.060
Hypertension	16,017 (45.2)	4,218 (44.6)	11,799 (45.4)	0.166
Joint pain	3,556 (10.0)	931 (9.8)	2,625 (10.1)	0.467
Fracture	631 (1.8)	204 (2.2)	427 (1.6)	0.001
Renal failure	696 (2.0)	167 (1.8)	529 (2.0)	0.104
Respiratory disease	1,877 (5.3)	562 (5.9)	1,315 (5.1)	0.001
Cerebrovascular disease	1,251 (3.5)	342 (3.6)	909 (3.5)	0.599
Living areas				<0.001
A	1,212 (3.4)	288 (3.0)	924 (3.6)	
B	860 (2.4)	232 (2.5)	628 (2.4)	
C	837 (2.4)	239 (2.5)	598 (2.3)	
D	1,049 (3.0)	277 (2.9)	772 (3.0)	
E	1,485 (4.2)	364 (3.8)	1,121 (4.3)	
F	1,949 (5.5)	518 (5.5)	1,431 (5.5)	
G	1,795 (5.1)	500 (5.3)	1,295 (5.0)	
H	970 (2.7)	265 (2.8)	705 (2.7)	
I	1,810 (5.1)	488 (5.2)	1,322 (5.1)	
J	1,981 (5.6)	556 (5.9)	1,425 (5.5)	
K	1,648 (4.7)	492 (5.2)	1,156 (4.5)	
L	1,961 (5.5)	512 (5.4)	1,449 (5.6)	
M	1,202 (3.4)	350 (3.7)	852 (3.3)	
N	2,128 (6.0)	593 (6.3)	1,535 (5.9)	
O	1,827 (5.2)	539 (5.7)	1,288 (5.0)	
P	2,147 (6.1)	544 (5.8)	1,603 (6.2)	
Q	2,260 (6.4)	555 (5.9)	1,705 (6.6)	
R	3,110 (8.8)	794 (8.4)	2,316 (8.9)	
S	1,654 (4.7)	413 (4.4)	1,241 (4.8)	
T	2,156 (6.1)	580 (6.1)	1,576 (6.1)	
U	1,384 (3.9)	357 (3.8)	1,027 (4.0)	
KCL domains				
ADL limitation	3,160 (8.9)	1,183 (12.5)	1,977 (7.6)	<0.001
Low physical strength	5,406 (15.3)	1,777 (18.8)	3,629 (14.0)	<0.001
Malnutrition	528 (1.5)	174 (1.8)	354 (1.4)	0.001
Oral dysfunction	7,280 (20.6)	2,148 (22.7)	5,132 (19.8)	<0.001
Isolation	3,163 (8.9)	1,154 (12.2)	2,009 (7.7)	<0.001
Memory decline	11,558 (32.6)	3,587 (37.9)	7,971 (30.7)	<0.001
Depressive mood	11,790 (33.3)	3,507 (37.1)	8,283 (31.9)	<0.001
Frailty status				
Robust (≤3 points)	13,031 (36.8)	2,956 (31.3)	10,075 (38.8)	
Prefrailty (4-7 points)	13,095 (37.0)	3,427 (36.2)	9,668 (37.2)	
Frailty (≥8 points)	9,299 (26.2)	3,073 (32.5)	6,226 (24.0)	

ADL: activities of daily living; IQR: interquartile range; KCL: Kihon Checklist; SD: standard deviation. Chi-square test; ^a^t-test. The names of living areas were anonymized and showed as alphabet letters.

The association of each domain of LTC risk and frailty status with nonresponse is shown in Table 2. The domains of ADL limitation (adjusted OR [aOR] 1.38, 95% CI 1.27-1.50), low physical strength (aOR 1.17, 95% CI 1.10-1.26), isolation (aOR 1.32, 95% CI 1.22-1.44), memory decline (aOR 1.23, 95% CI 1.17-1.30), and depressive mood (aOR 1.10, 95% CI 1.04-1.16) were associated with the nonresponse. The Spearman’s correlation coefficients between these seven domains of LTC risks were less than 0.5 for all pairs. Frailty status was associated with nonresponse in a dose-responsive manner (prefrailty, aOR 1.19, 95% CI 1.12-1.26; frailty, aOR 1.63, 95% CI 1.53-1.73).

**Table 2.** Associations of the KCL Domains and Frailty Status at Baseline (FY 2020 Survey) with Nonresponse to the Subsequent FY 2021 Survey.

KCL domain applicable	Unadjusted		Adjusted	
	OR (95% CI)	p Value	aOR (95% CI)	p Value
KCL domains^a^				
ADL limitation	1.74 (1.61-1.87)	<0.001	1.38 (1.27-1.50)	<0.001
Low physical strength	1.42 (1.34-1.52)	<0.001	1.17 (1.10-1.26)	<0.001
Malnutrition	1.36 (1.13-1.63)	0.001	1.15 (0.95-1.38)	0.152
Oral dysfunction	1.19 (1.13-1.26)	<0.001	1.04 (0.98-1.10)	0.226
Isolation	1.66 (1.54-1.79)	<0.001	1.32 (1.22-1.44)	<0.001
Memory decline	1.38 (1.31-1.45)	<0.001	1.23 (1.17-1.30)	<0.001
Depressive mood	1.26 (1.20-1.32)	<0.001	1.10 (1.04-1.16)	0.001
Frailty status				
Robust (≤3 points)	ref		ref	
Pre-frailty (4-7 points)	1.21 (1.14-1.28)	<0.001	1.19 (1.12-1.26)	<0.001
Frailty (≥8 points)	1.68 (1.58-1.79)	<0.001	1.63 (1.53-1.73)	<0.001

Multivariable logistic regression analyses are conducted after adjusting for age, sex, household income, chronic diseases, living areas, and individual KCL domains. ^a^All references to the seven KCL domains are “not at risk.” ADL: activities of daily living; aOR: adjusted odds ratio; CI: confidence interval; FY: fiscal year; KCL: Kihon Checklist; OR: odds ratio; ref: reference.

## Discussion

This population-based study of >30,000 community-dwelling older adults shows that those with ADL limitation, low physical strength, isolation, memory decline, depressive mood, and an overall frailty status were less likely to respond to the subsequent survey in the annual frailty screening program for LTC risks. These findings suggest that although the annual frailty screening program can be used to identify individuals at risk of LTC, nonrespondents were more likely to include those at high risk of LTC. Thus, they need to be surveyed through other methods, such as by providing certified mail or home-visit surveys.

The annual frailty screening program may be inefficient because individuals with LTC risks cannot be tracked through the survey. Our findings are consistent with previous findings that declines in ADL and physical, cognitive, and mental functions are associated with nonresponse in cohort studies ^(15), (16), (17)^. In addition to previously identified factors, isolation was found to be associated with nonresponse in the current study. A decrease in social activities, such as going outside, can cause functional decline ^(28), (29)^ and health-related issues ^(26)^, resulting in nonresponse. However, in this study, isolation itself was associated with nonresponse, even after adjusting for factors related to physical, memory, and other functions. Questionnaire responses can be considered a form of social interaction ^(16)^. Therefore, those in isolation might have had difficulty responding to the survey.

Older adults with frailty were less likely to respond. Previous studies have reported that older adults with higher levels of care needs are more likely to be nonresponsive to the mailed self-administered questionnaire ^(30)^. In addition, older adults with frailty are less likely to respond in a cohort study because of the deterioration of their health conditions or hospitalization ^(17)^. In the current study, some older adults with frailty might have difficulty responding because their health status had changed, even if they were not certified for care needs. Hence, older adults at high risk of frailty could constitute the target population for monitoring strategies.

From these results, we need to recognize that a nonresponse itself indicates the possibility of LTC risks. Additionally, selection bias should be considered when longitudinal studies are designed based on an annual frailty screening program, particularly if only complete cases are used in the analysis. Although conducting a home-visit survey among all nonrespondents is not feasible, municipalities need to find new approaches to improve the accuracy of the survey. For example, Ikoma City has implemented home visits by caregivers for follow-up of individuals who did not respond to the annual frailty screening program ^(31)^. The combination of multiple recruitment methods could be useful for improving the accuracy of indicators ^(16), (18)^. At the same time, it is important to invite individuals with LTC risks to preventive care services soon after their risks are identified, or it may be necessary to follow up with more active outreach. In outreach activities for older adults at higher risk of requiring LTC, comprehensive assessments are necessary based on their family structures, financial situation, and resources that they can or cannot utilize ^(32)^.

This study had some limitations. First, the current study included respondents in the FY 2020 survey, and thus, data from nonrespondents in the FY 2020 survey could not be obtained. We need to consider those who never responded to the annual frailty screening program, as they might be at a greater LTC risk than the study participants. Second, nonrespondents included more people who died after the baseline, but we could not exclude them because data on their deaths were unavailable. However, this effect was not directly assessed in the present study. We considered that this influence was limited because a previous study reported that the 6-month mortality rate among people requiring preventive care was low (1.2%) ^(33)^. Third, because the data were collected during the coronavirus disease 2019 (COVID-19) pandemic, social aspects such as isolation might have been affected. However, because we used data from FY 2020 and FY 2021 after the COVID-19 pandemic had already occurred, the situation at these two time points may not have differed substantially. Fourth, the FY 2021 survey included a higher number of questions than the FY 2020 survey, and this might have burdened the responders and affected the nonresponse rate. Particularly, the response rate decreased from 81.9% in the FY 2020 survey to 64.5% in the FY 2021 survey. The length of the questionnaires may have also been related to the nonresponse rate ^(18)^. Hence, it is possible that there were more individuals at LTC risks who did not respond to the subsequent survey. Finally, we used data from a single city in Japan, and thus, our findings may not be generalizable to other regions or countries. The municipality actively implemented LTC and welfare policies for older adults, but this may be different in municipalities with severe financial and human resource constraints. Despite these limitations, our results contribute to a better understanding of individuals at LTC risks and provide evidence of the importance of conducting outreach for nonrespondents, given that they are also at LTC risks.

In conclusion, the risk of LTC is associated with nonresponse to the annual frailty screening program in older adults in Japan. Although the program aimed to identify high-risk populations, older adults at LTC risk were less likely to respond to the survey. Thus, it may be necessary to modify the conventional survey methods used in the program or adopt a different approach to identify older adults with high LTC risks.

## Article Information

### Acknowledgments

The authors gratefully acknowledge the contributions of the municipal staff of Hachioji City.

### Author Contributions

Study concept and design: Kazumasa Nishida, Shinji Hattori, and Shota Hamada; data analysis and interpretation: Kazumasa Nishida, Reina Taguchi, Rumiko Tsuchiya-Ito, Tomoki Ishikawa, Satomi Kitamura, Masao Iwagami, Shinji Hattori, and Shota Hamada; drafting of the manuscript: Kazumasa Nishida, Reina Taguchi, Rumiko Tsuchiya-Ito, and Tomoki Ishikawa; critical revision of the manuscript for important intellectual content: Kazumasa Nishida, Reina Taguchi, Rumiko Tsuchiya-Ito, Tomoki Ishikawa, Satomi Kitamura, Masao Iwagami, Shinji Hattori, and Shota Hamada.

### Conflicts of Interest

Shota Hamada is an endowed chair, funded by donations from Hakue Technology, PROUMED, Japan BioProducts, Towa Pharmaceutical, Yellow Eight, and Sugi Holdings. Shota Hamada received research funding from SOMPO Care, Inc. outside of this work. The other authors have no conflicts of interest to declare.

### Approval by Institutional Review Board (IRB)

This study was approved by the institution affiliated with the first author (approval no. R5-001) and was conducted in accordance with the tenets of the Declaration of Helsinki.

### Data Availability Statement

All the relevant data for this study were obtained from the City of Hachioji. A contract exists between Hachioji City and the first author’s affiliated institution. The contract stipulates that Hachioji City will not allow the authors to provide the data to anyone other than the study members without permission from the city. Researchers interested in the data used in this study can contact the corresponding authors.

### Consent for Publication

Not applicable.

### Informed Consent

The requirement for informed consent was waived as this study used anonymous data collected for other purposes.

## Supplement

Supplementary Material
