# Supplementary material for: Association of Long-Term Care Risk with Nonresponse to the Annual Frailty Screening Program in Older Adults in Japan: A Retrospective Cohort Study
Source: JMA J. 2025 Sep 12;8(4):1184–91. doi: 10.31662/jmaj.2025-0092 (PMC12598306; doi:10.31662/jmaj.2025-0092)
Supplement: Supplementary Material [file 2433-3298-8-4-1184-s001.pdf]

Appendix 1. Differences in LTC risks between people certified as requiring LTC at baseline and study participants (nonrespondents and respondents)

	People who were certified as requiring LTC at baseline (n=176)	Nonrespondents (n=9,456)	Respondents (n=25,969)
	n (%)	n (%)	n (%)
ADL limitation	66 (37.5)	1,183 (12.5)	1,977 (7.6)
Low physical strength	86 (48.9)	1,777 (18.8)	3,629 (14.0)
Malnutrition	23 (13.1)	174 (1.8)	354 (1.4)
Oral dysfunction	60 (34.1)	2,148 (22.7)	5,132 (19.8)
Isolation	48 (27.3)	1,154 (12.2)	2,009 (7.7)
Memory decline	89 (50.6)	3,587 (37.9)	7,971 (30.7)
Depressive mood	107 (60.8)	3,507 (37.1)	8,283 (31.9)
Frailty status			
Robust (≤ 3 points)	25 (14.2)	2,956 (31.3)	10,075 (38.8)
Prefrailty (4–7 points)	39 (22.2)	3,427 (36.2)	9,668 (37.2)
Frailty (≥ 8 points)	112 (63.6)	3,073 (32.5)	6,226 (24.0)

ADL: activities of daily living.
